# A Role for Nanoparticles in Treating Traumatic Brain Injury

**DOI:** 10.3390/pharmaceutics11090473

**Published:** 2019-09-13

**Authors:** Badrul Alam Bony, Forrest M. Kievit

**Affiliations:** Department of Biological Systems Engineering, University of Nebraska-Lincoln, Lincoln, NE 68583-0726, USA; bbony2@unl.edu

**Keywords:** TBI, blood-brain barrier, nanomedicine, neurotrauma, nanotheranostics

## Abstract

Traumatic brain injury (TBI) is one of the main causes of disability in children and young adults, as well as a significant concern for elderly individuals. Depending on the severity, TBI can have a long-term impact on the quality of life for survivors of all ages. The primary brain injury can result in severe disability or fatality, and secondary brain damage can increase the complexities in cellular, inflammatory, neurochemical, and metabolic changes in the brain, which can last decades post-injury. Thus, survival from a TBI is often accompanied by lifelong disabilities. Despite the significant morbidity, mortality, and economic loss, there are still no effective treatment options demonstrating an improved outcome in a large multi-center Phase III trial, which can be partially attributed to poor target engagement of delivered therapeutics. Thus, there is a significant unmet need to develop more effective delivery strategies to overcome the biological barriers that would otherwise inhibit transport of materials into the brain to prevent the secondary long-term damage associated with TBI. The complex pathology of TBI involving the blood-brain barrier (BBB) has limited the development of effective therapeutics and diagnostics. Therefore, it is of great importance to develop novel strategies to target the BBB. The leaky BBB caused by a TBI may provide opportunities for therapeutic delivery via nanoparticles (NP). The focus of this review is to provide a survey of NP-based strategies employed in preclinical models of TBI and to provide insights for improved NP based diagnostic or treatment approaches. Both passive and active delivery of various NPs for TBI are discussed. Finally, potential therapeutic targets where improved NP-mediated delivery could increase target engagement are identified with the overall goal of providing insight into open opportunities for NP researchers to begin research in TBI.

## 1. Introduction

Traumatic brain injury (TBI) is a leading cause of death and disability worldwide, with approximately 2.87 million annual reported deaths, hospitalizations, and emergency room visits in the United States alone [1]. This is estimated to result in a $76.5 billion annual economic loss [2,3,4]. These substantial head injuries are caused by either a non-penetrating blow to the head, which results in bruising of the brain as well as tearing of axons, or a penetrating injury, which causes physical disruption to the brain. This primary injury is then followed by secondary injury, which can spread into the surrounding normal brain and is the target for therapeutic development. The adverse physiological change following a TBI is a complex process caused by calcium release, accumulation of reactive nitrogen species (RNS) and reactive oxygen species (ROS), glutamate toxicity, mitochondrial dysfunction, and neuroinflammation, which can lead to chronic progressive neurodegeneration [5,6,7,8,9]. The problem lies in a vicious positive feedback loop where primary physical damage to cells results in these biochemical derangements and damage-associated molecular patterns (DAMPS), which in turn leads to further cell death and the release of additional biochemical derangements and DAMPS [10,11]. Indeed, evidence of neuroinflammation has been observed up to 18 years post-injury [12], and chronic neuroinflammation is likely a driver of progressive neurodegeneration [13]. Moreover, there is increasing evidence of the role of secondary injury in chronic traumatic encephalopathy and other progressive neurodegenerative diseases [14,15,16,17]. This signifies these biochemical derangements as a primary driver of chronic secondary injury following a TBI.

The clinical management of TBI has progressed only incrementally and long-term injury is still a significant healthcare challenge. Currently, there is little evidence that supportive care therapies protect the surrounding brain. The spread of biochemical derangements into the surrounding brain is the primary concern to avoid secondary injury, which could reduce the spread of neuroinflammation and neurodegeneration. Indeed, many strategies that inhibit the effects of these biomolecules have shown promise in preclinical models and have been tested clinically, yet none have shown efficacy in the Phase III trial [18]. For example, the ProTECT trial sought to improve outcomes by reducing oxidative stress based on promising preclinical and early clinical data [19]. The compounds PEG-conjugated catalase (PEG-catalase), PEG-conjugated superoxide dismutase (PEG-SOD), and tirilazad have been used in free-radical scavenging. It is suggested, from preclinical studies, that progesterone has neuroprotective effects in brain injury models through multiple mechanisms, including modulating native antioxidant activity levels [20]. However, no improvement was found for other central nervous system (CNS) injuries treated with progesterone, and Phase III clinical trials have had limited success [21]. Cyclosporin A is thought to stabilize mitochondrial function in neurons to reduce the excitotoxic and oxidative stress that occurs in secondary damage, and it has shown promise in improving synaptic plasticity in rat models [22]. A phase IIa study has been completed with cyclosporin A, with plans for a larger phase II study (NeuroSTAT) [22].

The failures of recent clinical trials stem from an array of difficulties in treating a TBI (e.g., patient/biomarker selection, treatment timing, target engagement, etc.) [23], but from a fundamental science perspective there are two key targets for improvement, as follows: (1) Poor delivery to and retention in the brain, leading to limited therapeutic thresholds, and (2) off-target toxicity caused by many of the therapies targeting receptors of the biochemical derangements instead of the biochemical derangements themselves, which can result in loss-of-function in off-target cells. Therefore, there is a significant need to safely prevent the spread of biochemical derangements at the site of damage to prevent chronic progressive neuroinflammation and neurodegeneration.

Nanoparticles (NP) are uniquely suited to circumvent the formidable biological barriers that prevent transport and uptake of therapies into the brain. Thus, encapsulation of therapeutics within NPs is one of the approaches that can improve site-specific delivery, bioavailability, shelf-life, and also circumvent any potentially deleterious effects of the delivered therapeutics. The development of NPs for medical uses has been progressing for decades [24]. The vast majority of our knowledge of NP interaction with the human body, or mammalian organisms, comes from the significant efforts of cancer researchers. There is a substantial body of literature that exists which establishes a broad understanding of how the physicochemical and molecular properties of NPs affect their behavior in tumor tissues. For example, the enhanced permeability and retention (EPR) in tumors can be optimally exploited for passive NP targeting with NPs that have a hydrodynamic size of around 30 nm, while sizes between 10–100 nm still provide passive targeting. Additionally, alterations in surface charge and chemistry are known to play significant roles in NP behavior within the tumor. Cationic NPs remain on the exterior of tumors, but are readily taken up by cells, whereas anionic NPs distribute throughout the tumor because of the lack of interaction with the negatively charged extracellular matrix and cell membranes, but are poorly internalized [25]. This has led to the development of charge-reversal NPs that are initially negatively charged to promote distribution throughout the tumor, but then become positively charged once within the tumor microenvironment to promote cancer cell internalization [26,27,28]. Furthermore, active targeting strategies to increase the effectiveness of NPs were found not to increase accumulation at tumor sites, but did increase distribution throughout the tumor and promoted target cell internalization [29,30,31]. This was found to be caused by the EPR effect dominating NP accumulation at tumor sites. However, for micrometastases, where no EPR effect is apparent, active targeting is crucial for NP delivery [32]. Minimal uptake of NPs without targeting agents on their surface was observed in these micrometastases. Therefore, active NP targeted is necessary for accumulation in regions without an EPR effect. The knowledge base that has been generated on NP properties and their interaction with tumor tissue has resulted in multiple clinical trials exploring more effective treatment strategies suggesting NPs as potentially useful treatment vehicles.

However, the delivery of NPs into the central nervous system has remained a challenge due to the poor permeability of the blood-brain barrier (BBB) to NPs. Existing protocols have focused on the delivery of material locally, to either cerebrospinal fluid reservoirs or the brain tissue directly. Actively targeted NPs using the rabies virus glycoprotein (RVG)-targeting ligand have been used to specifically accumulate in neurons across the BBB and quiet a candidate therapeutic gene near the injured area [33]. Thus, the vast nanotechnology toolbox developed, in large part, by the efforts of cancer researchers can be utilized to establish NP-based techniques for active targeting strategies for gaining access to the diseased brain across the BBB.

## 2. Blood Brain Barrier and TBI 

The BBB is a restrictive barrier made up of tight junction proteins on brain endothelial cells that prevents the passive transport of blood components, including administered therapeutics, into the central nervous system (CNS). The BBB is part of an extensive network between endothelial cells, neurons, and glial cells which make up the neurovascular unit (NVU). Disturbances to the NVU caused by primary or secondary injury lead to the breakdown of the blood-brain barrier (BBB), which itself can further accelerate the progression of neurodegeneration through the leakage of neurotoxic molecules and reduced amyloid-β clearance. The BBB is periodically open within the first 24 h after TBI, thereby permitting the passage of substances, including macromolecules and neuroprotective drugs, that otherwise would be excluded from the brain [34]. The permeability of the BBB is partially controlled by inter endothelial junctions, which are protein complexes such as adherens junctions, tight junctions, and gap junctions [35]. Adherens junctions are protein complexes that occur at cell to cell junctions in epithelial and endothelial tissues and are usually located at more basal than tight junctions and control the permeability of the endothelial barrier. Tight junctions are multi-protein junctional complexes whose general function is to sustain the permeability barrier of epithelial and endothelial cells that control tissue homeostasis [36]. Gap junctions interconnect vascular cells homocellularly as well as providing heterocellular coupling between vascular smooth muscle cells and endothelial cells [37]. 

Following TBI, both immediate and delayed dysfunction of the BBB is observed. This reduction in function of tight junctions results in an increase in paracellular permeability. The post-traumatic oxidative stress, increased production of proinflammatory mediators, and upregulation of cell adhesion molecules of the brain endothelium surface can cause an increase in the influx of inflammatory cells into the injured brain parenchyma. Additionally, the expression of BBB associated transporters can be altered by a TBI, which may reduce functional interactions between the endothelium and glial cells and further reduce BBB function [38,39]. Indeed, post-traumatic changes to the functionality of the BBB seem to be a major driver of the progression of secondary injury and the extent of neuronal repair [40]. Nevertheless, disruption of the BBB provides an opportunity for passive NPs to target a damaged brain. 

## 3. Brain Delivery of NPs Across the BBB

NPs have been used in preclinical models of noncancerous neurological diseases in hopes of improving the circulation time of delivered therapeutics and enhancing delivery into the brain [41,42,43,44,45,46,47,48]. The BBB dysfunction enables blood-borne substances that are normally restricted to enter the brain parenchyma, either through increased paracellular leakiness or alterations in brain endothelial cell receptor expression, which may provide the opportunity for therapeutic delivery via NPs. The major target for therapeutic NP delivery is to provide neuroprotection and to reduce NVU disruption by preventing the spread of secondary injury beyond the primary contusional area. NP based applications have been more widely explored in the cerebral ischemia field as compared to TBI, yet NP transport across a disrupted BBB into these two brain pathologies could be similar. Transport into the brain can be achieved either passively or actively [49]. The passive transport routes indicate energy-independent processes, such as simple diffusion [50]. Moreover, the passive diffusion of drugs and NPs usually applies in tumor cells via the EPR effect [51]. Brain injury may lead to better BBB permeability, possibly due to tight junction disruption facilitated by injury-induced signaling milieu [52]. Under such conditions, the usual passage of impermeable molecules, as well as NPs, increases across the BBB [53]. Active transport, on the other hand, relies on energy-dependent cellular processes to bind to a target cell surface receptor, be internalized into the target cells, and then transported either to an intracellular site of action of a drug or transcellularly into the injured brain parenchyma.

Some of the first studies that addressed the ability of NPs to accumulate across a disrupted BBB in the damaged brain passively found an EPR-like effect present [33,54,55,56,57,58,59,60]. This work was followed by others that began to address how changes in NP size affects their accumulation across a disrupted BBB [58,61]. NPs around 100 nm accumulated at a higher level in the damaged brain as compared to larger NPs. At the other end of the scale, 3–5 nm dendrimers have been used to determine the nanoscale effects on access across a disrupted BBB in brain diseases [59,62,63,64,65,66], as well as other polymeric nanoparticle surface property effects in brain tissue and cellular distribution [57,61,64,67,68,69], which have provided a strong foundation for engineering desirable physicochemical properties of next-generation NPs. These studies found that the extent of dendrimer uptake is dependent on disease severity, the extent of BBB breakdown, and the level of glial cell activation. However, retention of these passively targeted NPs within the injured brain may be enhanced by a more active delivery approach by attaching targeting moieties (e.g., aptamers, peptides, antibodies, etc.) to the surface of the NP.

Various studies have begun to address how to achieve active targeting in noncancerous neurological diseases. Targeting agents included those that would increase NP uptake into neurons [33], normal brain vasculature [70,71,72,73,74,75,76], and mitochondria [77]. It is also essential to assess if brain uptake is affected by NP physicochemical properties. Findings of penetration within the brain parenchyma generally assume the NP remains stable within the brain environment. Very few studies have begun to address the stability of NPs within the brain microenvironment. Therefore, efforts have begun for better understanding the influence of size, concentration, and stability of the NPs in the brain microenvironment [67]. All of these factors could alter the function of NPs, such as diffusive capability and cellular uptake resulting in loss of utility as an effective therapeutic platform [78]. 

Several nanomaterials for TBI therapy have been studied and the section below highlights recent studies of passive and active targeting NPs for the treatment of TBI (Table 1). These mainly include antioxidant NPs that contain functional groups that inactivate ROS into less toxic species [5,56,60,79,80,81]. For example, NPs bound with superoxide dismutase (SOD) were shown to significantly reduce oxidative stress-mediated neuronal damage [82,83] and carbon NPs were shown to eliminate radical species in rat models of TBI [79]. NP-siRNA delivery vehicles were also used to protect neurons after a TBI by knocking down expression of the pro-apoptotic protein caspase 3 [33]. Various polyamidoamine dendrimer derivative nanoparticles, which have provided a robust framework for assessing nanoparticle size effects and drug delivery in improving recovery from TBI, [59,62,65,66] as well as other polymeric nanoparticle surface property effects in brain tissue and cellular distribution [57,61,64,67,68,69], have provided a strong foundation for engineering desirable physicochemical properties of next-generation NPs.

### 3.1. Lipid Based NPs

Liposomes were the first NP drug delivery system developed and are made of one or more vesicular bilayers (lamellae) that are composed of amphiphilic lipids with an internal aqueous compartment. Liposomes are biocompatible and can both trap and protect hydrophilic molecules in their internal water compartment as well as hydrophobic into the membrane. They also can provide a unique opportunity to deliver pharmaceuticals into cells or cellular compartments. Liposomes have been extensively utilized for brain drug delivery [87], including the treatment of cerebral ischemia [88] and brain tumors [89]. Solid lipid nanoparticles (SLN) contain a solid hydrophobic lipid core, which has been shown to be able to cross the BBB [90]. Modifying the surface properties of SLNs to include targeting ligands could improve delivery to the brain and limit RES uptake [91]. For example, Pluronic F-68 conjugated SLN could cause steric hindrance and decrease opsonization in plasma, which could delay the fast removal of particles from the reticuloendothelial system (RES) and extend the circulation time of SLN [92,93]. Wang et al. synthesized 3′,5-dioctanoyl-5-fluoro-2-deoxyuridine (DO-FUdR) to modify the limited access of the drug 5-fluoro-2,-deoxyuridine (FUdR), as well as its incorporation into SLN [94]. The results showed that DO-FUdR-SLN (76 nm) was able to deliver to the brain at twice the rate in vivo, as compared to free FUdR. This SLN could enhance the ability of the drug penetration through the BBB because of the increased retention of the DO-FUdR-SLN in the brain blood capillaries by adsorption to the capillary walls. This adsorption to the capillary walls resulted in a higher concentration gradient that enhanced the passage across the endothelial cell and delivery to the brain [94,95].

### 3.2. PBCA NPs

The first polymeric NP carrier to show drug delivery across the BBB were made of PBCA (poly butyl cyanoacrylate) [96]. PBCA NPs (350–450 nm) coated with polysorbate 80 were shown to be taken up more rapidly by endothelial cells [97]. Polysorbate 80 can absorb plasma apolipoprotein E (Apo-E) and then PBCA NPs (50 ± 5 nm) coated with Apo-E-bound polysorbate 80 are recognized as low-density lipoproteins (LDL), which are normally actively taken up by brain endothelial cells through receptor-mediated endocytosis [98]. Moreover, the BBB-penetrating properties of PBCA NPs have been exploited to transport numerous BBB-impermeable drugs across the BBB [98,99,100]. Voigt et al. investigated the effect of size (87 to 464 nm) and surfactant (neutral or cationic) on the ability of PBCA NPs to penetrate the BBB. They found that the surfactant (non-ionic or cationic) is the crucial factor determining BBB passage, rather than the size of nanoparticles [101]. Among polysorbate 80, Lutrol, Fluo-Tween, and Fluo-Lutrol, PBCA-NP with polysorbate 80 showed the highest passage across the BBB [101,102]. 

### 3.3. PLGA NPs

PLGA (poly(lactic-*co*-glycolic acid)) NPs loaded with cerebrolysin were synthesized, varying their sizes and surface properties, and applied both in normal and brain-injured rats. These PLGA NPs were able to reduce brain pathology following traumatic brain injury [42]. These cerebrolysin-loaded PLGA nanoparticles NPs (200 nm) were also able to reduce BBB breakdown most effectively 8 h following concussive head injury, as shown by Evans blue albumin and radioiodine, and the effect was most pronounced in the injured cortex as compared to the contralateral hemisphere. Cerebrolysin-loaded NPs showed therapeutic efficacy when administered even 4 h after TBI [42]. To use NPs to visualize the extent of the injury, PEG-coated 100 nm, 200 nm, and 800 nm PLGA NPs were synthesized and coupled to the 800 CW imaging agent, which can reveal the extent of the TBI by binding to intracellular proteins of cells that have lost membrane integrity [58]. It was found that 800 CW loaded 100 nm NPs can diffuse more deeply throughout the TBI, as compared to 800 CW loaded 800 nm NPs [58], indicating that smaller NPs should be more effective in drug delivery throughout the brain. It was also found that PLGA NPs coated with poloxamer 188 (PX) can be delivered into the brain of TBI mice with an improvement of neurological and cognitive deficits [41].

### 3.4. Dendrimer

Dendrimers are branched polymerics, similar to the structure of a tree. A dendrimer typically contains a symmetrical structure surrounding a central core, which forms a spheroidal morphology in water. Dendrimer NP-based delivery of therapeutics can increase accumulation in target tissue and reduce off-target side effects through similar control of size and surface properties described above. Albertazzi et al. showed that polyamidoamine (PAMAM) dendrimers (3.9 nm) were capable of diffusing into the CNS as well as penetrating into living neurons after intraventricular or intraparenchymal injections [103]. Kannan et al. prepared different sizes of PAMAM dendrimers (3.2, 3.9, 4.3, 6.7, 13.9, and 21 nm) for targeting neuroinflammation [62]. They found that the ability of the NPs to cross a disrupted BBB and accumulate in target glial cells was governed by BBB impairment and the level of glial cell activation. The cationic or anionic dendrimers neither escape the blood vessel nor extravasate into the tissue even after 24 h administration. The 4.3 nm neutral dendrimer (G4-OH) showed a 100-fold higher accumulation in the brain as compared to the free drug, showing the importance of zeta potential on brain delivery. Thus, dendrimer NP-mediated delivery offers significant opportunity for development of improved delivery strategies to TBI.

### 3.5. Gold NPs

Gold (Au) NPs are one of many metallic colloidal NPs types with tremendous applications because of their desirable properties, such as surface plasmon resonance, which can aid in imaging and biosensing. To improve Au NP targeting to endothelial cell membranes, PEG-coated Au NPs were synthesized and conjugated with a transactivator of transcription (TAT) peptide. However, both non-targeted and TAT targeted Au NPs (21.4 ± 0.9 nm) showed passive diffusion through the BBB, accumulating in both brain tumor and brain endothelial cells [104]. Additionally, Sela et al. found that 1.3 ± 0.3 nm Au NPs without surface modification could still penetrate the BBB in rats [105]. The Au NPs were uniformly distributed in both the hippocampus and the hypothalamus, indicating no binding selectivity of Au NPs in these regions of the brain. Further work by Setyawati et al. found 10 to 30 nm Au NP-induced micron-sized gaps among the endothelial cells after 30 min exposure, which were large enough to allow passive transport of drugs across the BBB [106]. This nanoparticle induced leakiness of endothelial cells (NanoEL) [106,107,108,109,110] provides a potential mechanism for heavy core NPs to penetrate the BBB. 

### 3.6. Silver NPs

Silver (Ag) NPs are another type of metallic NPs used in various fields [111]. It has been reported that Ag NPs (25 nm) could strongly interact with cerebral microvasculature and produce a proinflammatory cascade that resulted in BBB disruption, neuronal degeneration, and astrocyte reactivity [112]. However, inflammation and neurotoxicity caused by the Ag NPs to BBB at the cellular level are still unknown. To study the neurotoxicity resulted from Ag NPs (7 ± 2 nm) entering the brain, a triple co-culture BBB model of rat brain microvessel endothelial cells (rBMEC), pericytes, and astrocytes was established [113], which can also be engineered using induced pluripotent stem cells [114]. The altered protein and permeability of the BBB upon exposure to Ag NPs was investigated through TEM, where ultrastructural changes in pericytes, astrocytes, and endothelial cells were observed. Trickler et al. investigated the time-dependent effect of different sized (25, 40, and 80 nm) Ag NPs on the proinflammatory cascade in a rBMEC in vitro model [112]. From this study, it was found that the smaller Ag NPs showed larger effects on rBMEC at shorter time points as compared to larger Ag NPs, which may begin to explain a mechanism of NanoEL.

### 3.7. Silica NPs

Silica NPs are a promising candidate in biomedicine because of the availability of silica and the extraordinarily high surface areas of mesoporous silica NPs. Chen et al. synthesized various sizes of silica NPs (20, 40, and 80 nm) in order to find which penetrated the BBB most effectively [115]. The chemotherapeutic doxorubicin (DOX) was loaded into these NPs and then conjugated with cRGD peptide to improve its cancer-targeting effect. It was found that 40 nm DOX@Si NPs exhibit enhanced permeability across the BBB while simultaneously disrupting the ability of brain cancer cells to mimic vasculature [115]. Song et al. synthesized lactoferrin (Lf) conjugated silica NPs to test receptor-mediated delivery across the BBB. Among the three sizes (25, 50, and 100 nm) of NPs tested, the highest transport across the BBB was observed for lactoferrin conjugated 25 nm Si NPs [116], providing further evidence for the utility of smaller NPs in more effective delivery across the BBB. 

### 3.8. Carbon Dots

Carbon dots (CDs) are carbon-based NPs that have inherently tunable fluorescence and are non-toxic. Li et al. investigated transferrin conjugated CDs (9–12 nm) for the selective delivery of DOX to pediatric brain cancer cells because the transferrin receptor (TfR) can be overexpressed on the BBB as well as in cancer cells [117]. Lu et al. prepared CDs with a size of around 2.6 nm and with high quantum yield (51%) [118]. They hypothesized that CDs could cross the BBB because of their small size that could fit within tight junctions; however, this would also allow other small blood components to enter the brain, so the mechanism is more likely NanoEL or minor BBB disruption caused by tumor formation. The cytotoxicity of CDs was measured using 293 T cells and negligible cytotoxicity was observed after 24 h incubation. Moreover, 4 h following exposure, CDs showed high accumulation in the perinuclear region of the cells. To study the BBB-penetration ability of CDs, a biomimetic BBB model constructed from rBMEC and astrocytes were employed. It was found that the CDs were able to cross the BBB in a time and concentration-dependent manner, which was attributed to their small size [118]. Moreover, when cationic PEI was attached to the surface of the CDs, enhanced BBB penetration was observed; perhaps because of increased electrostatic interaction with the negatively charged cell membrane in this in vitro model [118].

These various different NP types provide an array of opportunities to attach different therapeutics including small molecule drugs, nucleic acids, and peptides. NPs that have a hydrophobic core, such as lipid-based, PLGA, and PBCA NPs, are typically better suited for small molecule delivery as compared to solid core NPs, which require chemical attachment to the NP surface. On the other hand, solid core NPs such as gold, silver, and silica have an advantage in peptide delivery, owing to the ease in surface modification such as silinization or coordination with thiol, hydroxyl, amine, or carboxyl groups. Nevertheless, each of these NP types could be used as effective vehicles to improve delivery into the damaged brain as long as their physicochemical properties are adequately controlled to ensure a near neutral charge for efficient delivery into the brain and they have a small size (<100 nm) to promote distribution throughout the injured tissue. However, the potential long-term retention of nanomaterials in the brain is a concern especially for solid core NPs that do not degrade. Therefore, translational studies that hope to bring nanomaterials into clinical trials should focus on NPs with known degradation profiles. Still, the use of solid core NPs will provide crucial information on NP-based strategies that will improve rapid and prolonged brain delivery.

## 4. Therapeutic Targets for NPs

The pathophysiological changes in the brain following a TBI have been well studied and characterized by new therapeutic targets that are still being identified. Nevertheless, these findings have yet to lead to an approved therapy for TBI, partially because of the lack of sufficient delivery into the brain and target engagement. Therefore, NPs that can deliver therapies targeting the pathophysiological changes may accelerate the translation of these basic science findings into widespread clinical use. There is currently a significant lack of NP-based strategies for treating the various pathophysiological changes following a TBI, which provides the opportunity for NP researchers to make an impact on the neurotrauma field. Table 2 summarizes numerous therapeutic targets that NPs could be designed to affect and exert a therapeutic benefit upon, many of which are unreported in the literature.

## 5. Conclusions

The understanding of mechanisms accounting for the long-term secondary progression of TBI has led to the discovery of numerous therapeutic targets. There is a significant need for local reduction in the biochemical derangements around the injured brain that give rise to the long-term progression of secondary injury. However, the clinical translation of TBI therapeutics has been hindered by limited delivery into, and especially retention in, the brain, which has extended opportunities for NP technology to improve target engagement. Notably, the role of NP-based systems to cross the BBB, which is periodically open within the first 24 h after TBI, and then be retained in damaged tissue is beginning to show promise in exerting a protective effect and preventing the spread of biochemical derangements to the surrounding healthy brain, potentially providing a significant advantage over other tested therapies that require continuous infusion. Furthermore, NP-based systems have shown promise in exploring novel approaches, such as detecting pre-inflammatory states to aid in early diagnosis. There are currently several challenges in the development of NPs for TBI where low reproducibility, poor scalability, and the high cost play a significant role in the lack of translation of nanotechnologies into clinical use. Therefore, scalable synthesis strategies that produce NPs with a high density of therapeutic materials, so that a relatively low concentration of the NP system remain effective, are needed to improve the clinical translation. Additionally, a neutral zeta potential has been shown to be critical in achieving brain delivery across a disrupted BBB, yet very few studies of NPs in TBI report surface charge. Therefore, a more thorough characterization of NP physicochemical properties should be included in future studies. An effective delivery vehicle would (1) greatly facilitate the evaluation of TBI therapeutics in animal models of injury and (2) facilitate translation of TBI therapeutics to human clinical trials. Furthermore, the multifunctionality of NPs provides a distinct advantage in being able to monitor accumulation kinetics, retention, and distribution, both in vitro and in vivo. Therefore, researchers must then be able to better understand the behavior of the NPs in the CNS so that therapeutics are maintained at a therapeutic level without rapid degradation. Success in the neurotrauma field may likely come from the implementation of nanotechnology to improve drug delivery and target engagement and may ultimately translate into clinical use to improve the survival and quality of life of TBI patients.

## Figures and Tables

**Table 1:** Summary of various nanoparticles for passive and active delivery for brain trauma applications ^a^.

NP Components	Therapeutic Mechanism	NP Size (nm)	NP Zeta Potential (mV)	Disease Model	Accumulation and Retention	Outcome	Ref.
Nanodrug with tetra ethylene glycol	α-tocopherol in the NP acts as an antioxidant and releases ibuprofen to reduce neuroinflammation	186	Not reported	Mice TBI: CCI	Accumulation shown after 36 h of intraperitoneal and intravenous injection	Behavior study showed significance for OFT (ambulatory) after IV injection, as compared to the saline group and not in IP.	[55]
Oxygen reactive polymer	Thioether group acts as antioxidant	8	Not reported	Mice TBI: CCI	Accumulated in damaged brain and retained for 18 h	Reduced neurodegeneration, astrogliosis, and activated microglia	[56]
Core-Cross-linked NPs	Thioether group acts as antioxidant	16	Not reported	Mice TBI: CCI	Accumulation shown within 1 h injection with retention for >2 h	Reduces intracellular ROS concentration in human astrocytes	[60]
PEGylated hydrophilic carbon cluster (PEG-HCC)	Oxygen radical annihilation at graphitic domains of the carbon particles.	50	Not reported	Rat mild TBI: (CCI)	6 h determine SO and NO levels	Restoration of CBF normalized oxidative radical profile (SO and NO levels)	[79]
PLGA NPs encapsulated with cerebrolysin	Cerebrolysin is a mixture of neuroprotective peptides	250–330	−13 mV	Mice TBI: stab wound	Not reported	Thwarts the edema formation at longer time points compared to bolus injection	[42]
PLGA NPs with/ without 800 CW coating	None	200	−39 mV	Mice TBI: cryolesion	Accumulation shown within 1 h of injection with retention for >48 h	NPs with 800 CW displayed preferential binding to intracellular proteins of cells that have lost membrane integrity	[72]
PLGA NPs coated with PX with BDNF encapsulation	BDNF acts as a neuroprotectant	170	Not reported	Mice TBI: closed head injury weight drop	Not reported	Significantly increased BDNF delivery and improved neurological and cognitive deficits	[41]
Polysorbate 80 PBCA NP HRP or EGFP	None	150	Not reported	Rat TBI: FPI	Not reported	HRP or EGFP delivered via PBCA NPs cross BBB and distributed near injury region	[84]
Porous silicon NPs conjugated with targeting peptide (CAQK) loaded with siRNA against GFP	Not reported	20	Not reported	Mice TBI: penetrating brain injury	Accumulated in brain for 2 h	Higher accumulation with 70% silencing of GFP expression	[85]
Targeted peptide from RVG, porous silicon NP graphene oxide (GO) coating with siRNA	None	190	+22.1 mV	Mice TBI: penetrating brain injury	Not reported	Increased (2.5-fold) delivery of siRNA via GO coated NPs compared to non-coating NPs	[86]

^a^ TEG (tetra ethylene glycol), CCI (controlled cortical impact), OFT (open field test), i.v. (intravenous), i.p. (intraperitoneal), PEG (polyethylene glycol), FPI (fluid percussion injury), MMP (matrix metallopeptidase), CBF (cerebral blood flow), NO (nitrate radical), PLGA (poly(lactic-co-glycolic acid)), PX (poloxamer 188), BDNF (brain derived neurotrophic factor), PBCA (polybutylcyanoacrylate), HRP (horse radish peroxidase), GFP (green fluorescent protein), EGFP (enhanced GFP), RVG (rabies virus glycoprotein).

**Table 2:** Therapeutic targets where NP-mediated delivery could provide an advantage including references where NPs against these targets have been tested in preclinical animal models.

Therapeutic Target	Pathophysiological Mechanism	Therapeutic NP Refs.
Reactive oxygen species	Increased oxidative stress leads to increased neurodegeneration and neuroinflammation.	[56,60,79]
Ischemia	Lack of oxygen delivery to injured brain leads to ischemic brain damage.	[119,120,121,122,123,124]
Mitochondrial dysfunction	Can increase oxidative stress and cell death in and around the injury.	[125]
BBB breakdown	Leads to accumulation of neurotoxic blood products and reduced function of the neurovascular unit.	[42]
Diffuse axonal injury	Neuronal membrane disruption leads to loss of axonal conduction and connections.	[126]
Neuroinflammation	Chronic activation of resident microglia and astrocytes as well as peripheral immune cell infiltration leads to an inflammatory milieu preventing recovery.	[63,66,127]
Neuroprotection	Direct protection of neurons from the dysregulated brain environment during secondary injury.	[33,41,42,43]
Lipid peroxidation products	A cascading event where oxidation of lipids leads to formation of lipid peroxidation products, which leads to further oxidation of lipids.	None
Glutamate	Release from necrotic cells leads to excitotoxicity in surrounding neurons.	None
Calcium	Release from necrotic cells leads to excitotoxicity in surrounding neurons.	None
